# Beyond Mistreatment at the Relationship Level: Abusive Supervision and Illegitimate Tasks

**DOI:** 10.3390/ijerph17082722

**Published:** 2020-04-15

**Authors:** Maie Stein, Sylvie Vincent-Höper, Marlies Schümann, Sabine Gregersen

**Affiliations:** 1 Department of Work and Organizational Psychology, University of Hamburg, 20146 Hamburg, Germany; sylvie.vincent-hoeper@uni-hamburg.de (S.V.-H.); marlies.schuemann@uni-hamburg.de (M.S.); 2 Institution for Statutory Accident Insurance and Prevention in the Health and Welfare Services, 22089 Hamburg, Germany; dr.sabine.gregersen@bgw-online.de

**Keywords:** abusive supervision, hostility, task-related supervisory behavior, illegitimate tasks, unnecessary tasks, unreasonable tasks, hierarchical level

## Abstract

According to the concept of abusive supervision, abusive supervisors display hostility towards their employees by humiliating and ridiculing them, giving them the silent treatment, and breaking promises. In this study, we argue that abusive supervision may not be limited to mistreatment at the relationship level and that the abuse is likely to extend to employees’ work tasks. Drawing upon the notion that supervisors play a key role in assigning work tasks to employees, we propose that abusive supervisors may display disrespect and devaluation towards their employees through assigning illegitimate (i.e., unnecessary and unreasonable) tasks. Survey data were obtained from 268 healthcare and social services workers. The results showed that abusive supervision was strongly and positively related to illegitimate tasks. Moreover, we found that the relationship between abusive supervision and unreasonable tasks was stronger for nonsupervisory employees at the lowest hierarchical level than for supervisory employees at higher hierarchical levels. The findings indicate that abusive supervision may go beyond relatively overt forms of hostility at the relationship level. Task-level stressors may be an important additional source of stress for employees with abusive supervisors that should be considered to fully understand the devastating effects of abusive supervision on employee functioning and well-being.

## 1. Introduction

Healthcare and social services workers frequently become the target of verbal and nonverbal abuse in their work environment [1,2]. While they may experience abusive behaviors from several different perpetrators (e.g., patients/clients and their relatives, coworkers), an important source of abuse that should not be overlooked is the supervisor [3]. Abusive supervisors humiliate and ridicule their employees, break promises, yell and scream, and purposely withhold needed information [4]. This expression of hostility is a significant stressor for employees that has deleterious effects on different aspects of employee functioning and well-being, such as anxiety, depression, psychosomatic symptoms, and emotional exhaustion [5,6,7].

However, supervisory abuse may not be limited to the relatively overt hostility at the relationship level. Given that supervisors play a key role in defining work roles and assigning tasks [8], abusive supervision is likely to extend to employees’ work tasks [9]. In particular, abusive supervisors may display disrespect and devaluation towards their employees through the tasks they assign to them. One way through which abusive supervisors may send an implicit message of disrespect and devaluation is the assignment of illegitimate tasks, which refer to tasks that are unnecessary (i.e., tasks that are pointless and serve no meaningful purpose) or unreasonable (i.e., tasks that fall outside one’s job role expectations) and that could and should have been prevented from being assigned [10]. 

**Figure 1:**
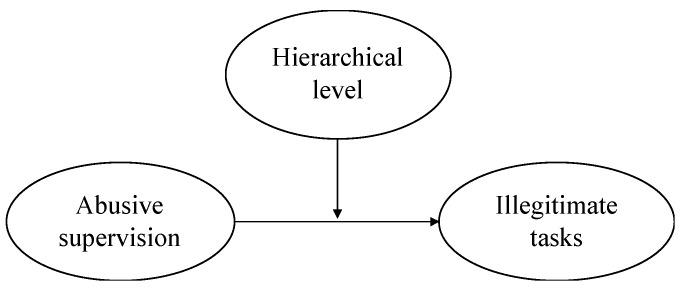
The conceptual model.

In this study, we recognize that abusive supervisors’ displays of hostility may manifest in their employees’ task assignments and investigate the relationship between abusive supervision and employees’ illegitimate (i.e., unnecessary and unreasonable) work tasks. Furthermore, we aim to shed light on the moderating role of the wider context in which the abusive supervision occurs. Specifically, we argue that the relationship between abusive supervision and illegitimate tasks varies depending on the hierarchical level and propose that the relationship between abusive supervision and illegitimate tasks might be stronger for nonsupervisory employees than for employees with supervisory responsibility. Figure 1 shows the conceptual model. 

This study contributes to the literature on abusive supervision by incorporating task-related abusive supervisory behaviors. Specifically, we shift the focus from relatively direct messages of disrespect and devaluation in interpersonal interactions to less overt forms of supervisory abuse at the task level. To fully understand the various detrimental effects of abusive supervision on employees, it is crucial to recognize that abusive supervision may cause harm through employees’ task assignments. Adding a task-related perspective to complement the concept of abusive supervision may advance the understanding of alternative mechanisms for explaining the harmful effects of abusive supervision on employee functioning and well-being. In particular, this perspective sets the stage for investigating explanatory mechanisms beyond employees’ efforts to cope with the socioemotional stressors arising from supervisory mistreatment at the relationship level. 

Moreover, we refine the understanding of the role of the wider organizational context in which abusive supervision occurs [11]. Previous research has mainly focused on how contextual factors (e.g., work unit structure, power distance) moderate the relationships between abusive supervision and its antecedents and consequences [6,12]. By considering the moderating role of hierarchical level, we acknowledge that the forms of supervisory abuse in themselves might vary depending on the employee’s position in the organization and further theorizing about what other differences in the manifestation of abusive supervision across organizational levels might exist. 

## 2. Theoretical Background and Hypotheses

### 2.1. Abusive Supervision

The dark side of supervisory behavior in organizations has received increasing attention over the past decade [11]. Abusive supervision is the concept that has been most frequently discussed in studies investigating destructive forms of leadership within an organization [7]. According to Tepper’s [4] conceptualization, abusive supervision refers to employees‘ perceptions of the extent to which their supervisor engages in sustained displays of verbal and nonverbal hostility, excluding physical contact. Examples of abusive supervisory behavior include being rude, breaking promises, humiliating employees in front of others, invading employees’ privacy, wrongly blaming employees, and purposely withholding information [5]. Abusive supervisors act willfully, meaning that they perpetrate abusive behavior for a purpose, but their immediate intent is not necessarily to cause harm. While harmful intent is an important motive of abusive supervision, abusive supervisors may mistreat their employees to accomplish objectives other than causing harm (e.g., to motivate high performance) [5]. 

Regardless of their motives, abusive supervisors display little concern for the welfare of their employees [9], and the experience of abusive supervision is highly stressful for employees [4]. A considerable body of research indicates that abusive supervision has deleterious effects on several aspects of employee functioning and well-being. For example, abusive supervision has been found to be positively related to anxiety, depression, psychosomatic symptoms, and emotional exhaustion [5,6,7]. Research has provided several explanations for these findings, which mainly center around the notion that abusive supervision is harmful due to the socioemotional stressors that arise from supervisory mistreatment at the relationship level [13,14,15]. However, socioemotional stressors arising from personal forms of mistreatment may not be the only source of stress for employees with abusive supervisors. We argue that abusive supervision is likely to manifest in mistreatment at the task level, thus creating an additional source of stress for employees beyond the relationship level.

### 2.2. Abusive Supervision and Illegitimate Tasks

In their “root cause” framework of poor leadership, Kelloway and colleagues [9] state that poor leadership is not only stressful in and of itself but may also give rise to other occupational stressors. Since one of the core responsibilities of supervisors is to assign tasks to employees [8], it seems likely that these additional stressors arise at the task level. Consistent with this idea, Rodwell and colleagues [16] noted that abusive supervision may not only comprise “personal attacks” (e.g., displays of ridicule, rudeness, and lies) but also “task attacks” (e.g., excessive monitoring). 

For positive supervisory behavior (e.g., transformational leadership), several empirical studies have demonstrated favorable effects on the characteristics of employees’ work tasks [17,18,19]. However, knowledge on the task-related behavior of abusive supervisors is very limited. While Rodwell and colleagues [16] provide convincing theoretical arguments for an association, they did not test how supervisors’ “task attacks” relate to the overall concept of abusive supervision. One notable exception that empirically brings together abusive supervision and task-related stressors originating from supervisors is the work of Wu and colleagues [20]. In their study, they examined the association between abusive supervision and workload demands from supervisors (e.g., placing unrealistic/unfair demands on employees) and investigated how these two types of supervisor-related stressors differentially relate to employees’ levels of emotional exhaustion. The findings of this study show that abusive supervision and workload demands imposed by supervisors are two closely related yet distinct stressors. However, the link between abusive supervision and workload demands from the supervisor is not self-explanatory because high levels of workload that come from the supervisor may not necessarily imply disrespect and devaluation but are often inevitable due to organizational constraints. 

For illegitimate tasks, the association with abusive supervision is more straightforward. Rooted in stress-as-offense-to-self (SOS) theory [21], illegitimate tasks are a relatively new concept of occupational stressor that exists at the task level [10,22]. The concept of illegitimate tasks involves unnecessary and unreasonable tasks. Unreasonable tasks refer to those that are perceived to fall outside one’s job role requirements, implying a violation of the person’s role and status. Tasks are not inherently unreasonable but become unreasonable in the context of specific job roles and situations [22]. For example, mopping the ward floor is likely an unreasonable task for certified nurses who are very busy caring for patients. Unnecessary tasks refer to tasks that could have been avoided or performed with reduced effort through a different way of organizing. For example, employees may feel that filling in the same data into two different lists that could easily be combined into one list is an unnecessary task. 

An illegitimate task sends an implicit message of disrespect, devaluation, and carelessness [22], and “the label “illegitimate” suggests that someone could and should have prevented it from being assigned” [23] (p. 765). Given that the expressions of disrespect, devaluation, and carelessness are core components of abusive supervision, it seems likely that the behavioral repertoire of abusive supervisors comprises the assignment of illegitimate tasks as an additional form of supervisory abuse. While the original conceptualization of abusive supervision [4] does not refer to this form of behavioral abuse, we suggest that abusive supervisors may carelessly and/or intentionally assign employees unnecessary and unreasonable tasks, thus expressing disrespect and devaluation. This perspective fits well with the notion that abusive supervisors display little concern for their employees’ welfare in all their actions [9]. Therefore, we hypothesize the following:

**H1.**  *Abusive supervision is positively related to (a) unnecessary tasks and (b) unreasonable tasks.*

### 2.3. The Role of Hierarchy Level

Supervisors at different hierarchical levels of an organization have fundamentally different tasks, responsibilities, and opportunities [24]. One important difference lies in the degree to which supervisors at different organizational levels are involved in their employees’ activities. While first-level supervisors are commonly responsible for planning and assigning work tasks, supervisors at higher organizational levels establish higher-order operational goals and set strategic objectives for the overall organization [25]. Given the specific role requirements of first-level supervisors (i.e., planning and assigning tasks), it seems likely that first-level abusive supervisors might more frequently assign illegitimate tasks to their employees (i.e., those at the lowest organizational level) than abusive supervisors at higher organizational levels who, in general, assign fewer tasks to their employees.

Moreover, employees in supervisory positions generally have more control over their work contents and the tasks they must perform than nonsupervisory employees. Through their higher levels of control, they might be able to prevent themselves from performing illegitimate tasks. On the one hand, supervisory employees might have the power to decide whether any illegitimate tasks they have been assigned may be done differently (e.g., in a more meaningful way) or not at all. On the other hand, supervisors might have the opportunity to pass down their illegitimate tasks to employees at the subordinate level. This notion is consistent with research on the trickle-down effects of abusive supervision, showing that abusive behavior trickles down across organizational levels [26,27]. 

Taken together, these arguments suggest that the association between abusive supervision and illegitimate tasks might be stronger for employees at the lowest hierarchical level than for employees at higher hierarchical levels of an organization. In other words, nonsupervisory employees (i.e., those at the lowest organizational level) might more frequently experience assignments of illegitimate tasks from their abusive supervisor than employees with supervisory responsibility (i.e., those at higher organizational levels). Therefore, we hypothesize the following:

**H2.**  *Hierarchical level moderates the relationship between abusive supervision and (a) unnecessary tasks and (b) unreasonable tasks such that the relationships are stronger for nonsupervisory employees (i.e., those at the lowest organizational level) than for supervisory employees (i.e., those at higher organizational levels).*

## 3. Materials and Methods 

### 3.1. Participants and Procedure

The study received approval from the Local Ethics Committee of the Faculty of Psychology and Human Movement at the Universität Hamburg (no. 2017_144). Using an online survey study design, we collected data from 268 healthcare and social services workers. Participants were recruited through a panel management and online research company. The requirements for participation included being currently employed and having a direct supervisor. A total of 78% of the participants were female. The mean age was 45.6 years (*SD* = 11.24), and the mean professional tenure was 16.37 years (*SD* = 11.75). The mean working hours per week was 32.15 hours (*SD* = 10.22). Nearly one-third (32%) of the participants held a supervisory position. A total of 18% of the participants had worked with their supervisor for less than one year, 40% had worked with their supervisor for one to five years, and 42% had worked with their supervisor for more than 5 years.

### 3.2. Measures

We measured abusive supervision with 12 items from Tepper‘s [4] scale, which has been shown to be a reliable and valid measure for assessing abusive supervisory behavior [4,28,29]. A sample item is “My supervisor puts me down in front of others.” Items were scored on a five-point Likert scale ranging from 1 (“never”) to 5 (“very often”). Cronbach’s alpha was α = 0.95. Illegitimate tasks were measured using 8 items from the Bern Illegitimate Tasks Scale (BITS), which assesses unnecessary and unreasonable tasks with 4 items each [10]. Sample items include “Do you have work tasks to take care of that keep you wondering if they just exist because some people simply demand it this way?” for unnecessary tasks and “Do you have work tasks to take care of that you believe should be done by someone else?” for unreasonable tasks. Responses were scored on a five-point Likert scale ranging from 1 (“never/rarely”) to 5 (“very often”). Several studies provide evidence for the validity and reliability of this scale [10,22,30]. Cronbach’s alphas were α = 0.90 for both unnecessary tasks and unreasonable tasks. Hierarchical level was assessed by asking the participants whether they held a formal supervisory position (0 = no, 1 = yes).

### 3.3. Statistical Analyses

To test the measurement models of abusive supervision and illegitimate tasks, we conducted confirmatory factor analysis (CFA) in R version 3.6.2 [31] using the lavaan package [32]. Items were used as indicators of the respective latent factors, and the model parameters were obtained using robust maximum likelihood estimation. To assess model fit, we computed χ^2^ statistics. Nonsignificant χ^2^ values indicate that the model fits the data well. Because the χ^2^ statistics are sensitive to sample size, additional fit indices were considered: the comparative fit index (CFI), the squared root mean residual (SRMR), and the root mean square error of approximation (RMSEA). General guidelines suggest that values close to 0.95 or higher for CFI, levels of 0.08 or lower for SRMR, and levels of 0.06 or lower for RMSEA indicate adequate fit [33]. 

To test the hypotheses, we computed linear ordinary least squares (OLS) regression models in R. Abusive supervision, professional tenure, and working hours were centered at their respective means to facilitate the interpretation of the coefficients. The interaction term was built by multiplying the values of abusive supervision and hierarchical level together. In testing the moderator hypotheses, we included the independent variables along with the interaction term in the regression models. 

### 3.4. Control Variables

We decided to include several control variables in the models. First, we controlled for gender, because evidence exists that women might perform more illegitimate tasks than men [30]. Second, we controlled for tenure, because it has been suggested that those with longer tenure perceive less abusive behavior [14], and it is also conceivable that they perceive fewer illegitimate tasks. Third, we controlled for working hours per week, because it has been argued that employees who work more hours might be assigned more illegitimate tasks [34]. 

## 4. Results

### 4.1. Measurement Models

The three-factor CFA model, in which the items of abusive supervision and unnecessary and unreasonable tasks loaded onto their respective latent factors, yielded a good fit with the data (χ^2^(167) = 367.90, *p* < 0.001; CFI = 0.93; RMSEA = 0.080; SRMR = 0.046). The fit of the two-factor model, in which the items of unnecessary and unreasonable tasks loaded onto one latent factor and the items for abusive supervision loaded onto another latent factor, was not satisfactory (χ^2^(169) = 478.74, *p* < 0.001; CFI = 0.90; RMSEA = 0.093; SRMR = 0.050). Thus, although the latent factors of unnecessary and unreasonable tasks were highly correlated (*r* = 0.81), we found evidence for the notion that unnecessary and unreasonable tasks should be considered distinct constructs. Therefore, we followed previous studies [35,36] and examined the two forms of illegitimate tasks separately.

### 4.2. Descriptive Statistics

Table 1 shows the means, standard deviations, and correlations of the study variables. Of the control variables, gender was negatively correlated with working hours per week (*r* = −0.25, *p* < 0.001) and hierarchical level (*r* = −0.14, *p* = 0.026). Professional tenure was not related to any of the study variables. Working hours per week were positively related to hierarchical level (*r* = 0.26, *p* < 0.001), abusive supervision (*r* = 0.22, *p* < 0.001), unnecessary tasks (*r* = 0.22, *p* < 0.001), and unreasonable tasks (*r* = 0.22, *p* < 0.001). Hierarchical level showed a small positive correlation with unreasonable tasks (*r* = 0.12, *p* = 0.048). In line with Hypothesis 1, abusive supervision was positively related to unnecessary tasks (*r* = 0.52, *p* < 0.001) and unreasonable tasks (*r* = 0.52, *p* < 0.001). 

### 4.3. Hypothesis Testing

Table 2 displays the results of the regression analyses for testing the relationship between abusive supervision and illegitimate tasks and the moderating effect of hierarchical level. The results showed that abusive supervision was positively related to unnecessary tasks (*B* = 0.64, *p* < 0.001) and unreasonable tasks (*B* = 0.62, *p* < 0.001), providing support for Hypothesis 1.

**Figure 2:**
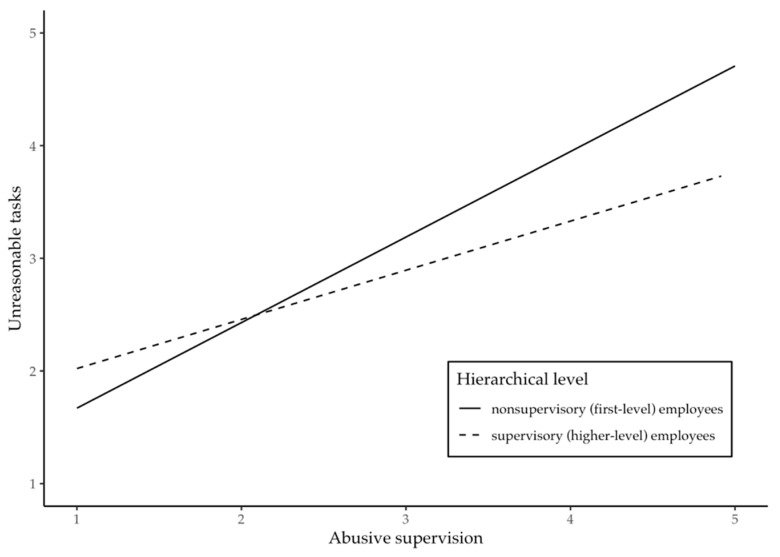
The moderating effect of hierarchical level on the relationship between abusive supervision and unreasonable tasks.

Contrary to Hypothesis 2a, we did not find a significant moderating effect of hierarchical level on the relationship between abusive supervision and unnecessary tasks (*B* = −0.16, *p* = 0.27). However, we found a significant moderating effect of hierarchical level on the relationship between abusive supervision and unreasonable tasks (*B* = −0.32, *p* = 0.022). In line with Hypothesis 2b, hierarchical level moderated the relationship between abusive supervision and unreasonable tasks such that abusive supervision was more strongly related to unreasonable tasks for nonsupervisory employees at the lowest hierarchical level than for supervisory employees at higher hierarchical levels. Figure 2 shows the form of the interaction. 

## 5. Discussion

Drawing upon the “root cause” framework of poor leadership [9], we argued that abusive supervisors’ displays of hostility are likely to extend to their employees’ work tasks. In particular, we suggested that illegitimate (i.e., unnecessary and unreasonable) tasks might be an additional form of abusive supervisory behavior that signals disrespect and devaluation to employees and proposed that abusive supervision is positively related to employees’ levels of unnecessary and unreasonable tasks. The results revealed strong positive relationships between abusive supervision and employees’ unnecessary and unreasonable tasks. Moreover, we found that hierarchical level moderated the relationship between abusive supervision and unreasonable tasks. For nonsupervisory employees (i.e., those at the lowest hierarchical level), abusive supervision was more strongly related to unreasonable tasks than for employees with supervisory responsibility (i.e., those at higher hierarchical levels). For unnecessary tasks, we did not find a moderating effect of hierarchical level. 

### 5.1. Theoretical Implications

The findings of this study provide support for the notion that abusive supervision is not limited to relatively overt forms of hostility that occur at the relationship level but is also associated with task-level stressors [16]. Theoretically, abusive supervision focuses on mistreatment from the supervisor at the relationship level [5]. Although this focus on mistreatment at the relationship level is intentional, this study suggests that abusive behavior at the relationship level should not be seen separately from task-level stressors originating from abusive supervisors. Excluding abuse at the task level would mean neglecting an important additional source of stress for employees with abusive supervisors [20]. To fully understand the devastating impact of abusive supervision on employees, the relationship-level perspective on supervisory abuse [5] needs to be complemented by abusive supervisory behavior targeted at the employees’ work tasks [20]. 

The “root cause” framework of poor leadership [9] states that “the presence, absence, or intensity of particular stressors may be determined by the quality of leadership in the workplace (p. 95). In particular, poor leadership is not only a stressor in and of itself but also gives rise to other occupational stressors. Empirical research utilizing this framework has focused on supervisors’ influence on their employees’ traditional role stressors [37,38]. We complement the “root cause” framework by showing that the behavioral repertoire of abusive supervisors also includes the assignment of illegitimate tasks. Although illegitimate tasks may be considered a specific form of role conflict, they add a qualitatively different dimension to role stress because they threaten the identity of the receiver of the abuse and elicit strong feelings of injustice [22]. Thus, paying attention to the assignment of illegitimate tasks contributes to a better understanding of how abusive supervisors shape a stressful work environment for their employees.

Closely related to abusive supervision, the concepts of petty tyranny [39] and supervisor undermining [40] comprise different forms of task-related supervisory mistreatment. Petty tyranny includes relatively broad descriptions of “tyrannical” supervisory behaviors related to employees’ work, such as eliminating consideration and discouraging initiative [39]. However, unlike abusive supervision, petty tyranny may not necessarily be viewed as hostile [5]. Supervisor undermining refers to supervisor “behavior intended to hinder, over time, the ability to establish and maintain positive interpersonal relationships, work-related success, and favorable reputation” [40] (p. 332). For example, supervisor undermining includes a supervisor’s deliberate and intentional efforts to delay his or her employees’ work to make them look bad or slow them down. However, the perception of harmful intent, which is not a definitive characteristic of abusive supervision [5], is a core aspect of supervisor undermining. Nonetheless, despite these conceptual differences, petty tyranny and social undermining may guide theorizing about the existence of other task-related forms of abusive supervisory behavior beyond illegitimate tasks and workload demands [20]. 

Moreover, the findings of this study suggest that the form of abusive supervision may differ across organizational levels. Specifically, we found that abusive supervision was more strongly related to unreasonable tasks for nonsupervisory employees than for employees with supervisory responsibilities. For unnecessary tasks, we did not find a moderating effect of hierarchical level. This finding might be related to the differences in the extent to which supervisory employees may pass down their unnecessary and unreasonable tasks. In particular, an unreasonable task is unreasonable for a specific employee but may fit another employee’s job description [41]. Thus, supervisory employees may release themselves from unreasonable task assignments from their abusive supervisor by delegating the tasks to a subordinate employee for whom the task may not be illegitimate. Given that there are no employees subordinate to them, nonsupervisory employees with abusive supervisors may not have this opportunity to delegate an unreasonable task to another, more suitable employee.

In contrast, unnecessary tasks are unnecessary, regardless of the organizational level at which the tasks occur. Since unnecessary tasks should actually not be performed at all, supervisory employees who are assigned unnecessary tasks from their abusive supervisor may therefore refrain from passing down these tasks, which may explain why we found no differences between supervisory and nonsupervisory employees in the associations between abusive supervision and unnecessary tasks. However, this explanation is speculative and has yet to be tested.

### 5.2. Practical Implications

The results of this study strengthen the need for organizations to reduce abusive supervision to avoid the far-reaching negative consequences of abusive supervisory behavior. When identifying abusive supervision in the workplace, organizations may not only focus on “traditional” forms of abuse at the relationship level such as yelling and humiliating. Rather, organizations should also pay attention to the assignment of illegitimate tasks as a less overt form of abusive supervisory behavior. Given that the results suggest that abusive supervision is more strongly related to unreasonable tasks for nonsupervisory employees than for employees with supervisory responsibility, this recommendation might be even more important at the lowest organizational level. 

The findings may also be incorporated into training programs that aim to reduce abusive supervision in organizations. Such training should not only convey knowledge on how to prevent behavioral mistreatment at the relationship level but should also sensitize supervisors to the fact that illegitimate tasks are a significant source of stress for employees. Supervisors should be trained to consider the potential illegitimacy of the tasks they assign and to express respect in another way if an illegitimate task is inevitable. 

### 5.3. Limitations and Future Directions

Several limitations should be considered when interpreting the findings. First, we cannot draw conclusions about the causality of effects. It is conceivable that the employees’ levels of illegitimate tasks influence their perception of the supervisor. Although abusive supervision comprises behaviors that are clearly abusive (e.g., yelling and screaming) and it seems less likely that employees with high levels of illegitimate tasks would perceive nonabusive supervisory behaviors as abuse, we cannot rule out the fact that the assignment of illegitimate tasks might result in employees perceiving higher levels of abusive behaviors from their supervisors. Therefore, we recommend future research to address the causality issue by using longitudinal and experimental study designs.

Second, the use of self-report measures raises concerns about common method variance [42]. However, given that abusive supervision and illegitimate tasks refer to subjective perceptions, the focal person might be the most appropriate source of assessment. Importantly, the views of the focal person and other raters (e.g., supervisors, coworkers) may not necessarily converge [23]. Nonetheless, to strengthen the findings, further research should try to collect data from different sources, such as by incorporating coworkers’ perspectives. In addition, introducing a time lag between the measurements may also help reduce the potential bias due to common method variance [42].

Furthermore, the mechanisms explaining why abusive supervision is related to illegitimate tasks remain unclear. While it is conceivable that abusive supervisors intentionally assign illegitimate tasks to cause harm, high levels of illegitimate tasks may also be the result of the abusive supervisor’s poor supervisory skills (e.g., abusive supervisors’ lack of awareness of or concern for the consequences of their behavior) [9]. In addition, supervisors may have performance motives for their actions and (carelessly) assign illegitimate tasks to get urgent tasks done.

On a related note, it is important to recognize that illegitimacy is not inherent in tasks [22]. Rather, tasks become illegitimate in a specific context. Previous research has shown that supervisors’ framing (e.g., acknowledgment) may mitigate employees’ perceptions of illegitimacy [43]. Such framing effects may also occur in the context of abusive supervision. The fact that a task is assigned by an abusive supervisor might increase the extent to which employees perceive this task as illegitimate. Thus, the close association between abusive supervision and illegitimate tasks may partly be a perceptual phenomenon. Nonetheless, the stress originating from the assignment of illegitimate tasks is real and likely to impair employee functioning and well-being. 

Finally, in terms of future research, it may be worthwhile to take a closer look at the causes and mechanisms for explaining why the assignment of unreasonable tasks is more strongly related to abusive supervision for nonsupervisory employees than for employees with supervisory responsibility. Theoretically, we have offered several explanations for this difference, which center around the notion that supervisors at different organizational levels have different tasks, responsibilities, and opportunities. To obtain a better understanding of why the forms of supervisory abuse differ across organizational levels, we recommend future studies to test these explanations.

## 6. Conclusions

In this study, we investigated the relationship between abusive supervision and employees’ illegitimate tasks. Drawing upon the notion that one of the key tasks of supervisors is to assign work tasks, we argued that one straightforward way through which abusive supervisors display disrespect and devaluation to their employees is the assignment of illegitimate (i.e., unnecessary and unreasonable) tasks. The results revealed strong associations between abusive supervision and employees’ levels of unnecessary and unreasonable tasks. In addition, we found that the relationship between abusive supervision and unreasonable tasks was stronger for nonsupervisory employees (i.e., those at the lowest hierarchical level) than it was for supervisory employees at higher hierarchical levels. To obtain a complete picture of the deleterious effects of abusive supervision on employees’ functioning and well-being, it is important to recognize that abusive supervision is not limited to the relationship level but is likely to extend to employees’ work tasks, giving rise to additional sources of stress.

## Figures and Tables

**Table 1:** Means, standard deviations, and correlations of the study variables.

Variable	*M*	*SD*	1	2	3	4	5	6
1 Gender ^1^	0.78	0.40						
2 Tenure	16.37	11.75	−0.01					
3 Working hours	32.15	10.22	−0.25 ***	0.06				
4 Hierarchical level ^2^	0.32	0.47	−0.14 *	0.07	0.26 ***			
5 Abusive supervision	1.56	0.75	0.08	0.06	0.22 ***	0.09		
6 Unnecessary tasks	2.54	0.98	0.00	−0.02	0.22 ***	0.09	0.52 ***	
7 Unreasonable tasks	2.14	0.95	0.04	0.02	0.22 ***	0.12 *	0.52 ***	0.74 ***

Note: *N* = 268. Pearson correlation coefficients. ^1^ 0 = male; 1 = female. ^2^ 0 = nonsupervisory employees (i.e., lowest hierarchical level); 1 = supervisory employees (i.e., higher hierarchical levels). * *p* < 0.05; *** *p* < 0.001.

**Table 2:** Results of the regression analyses.

	Unnecessary Tasks			Unreasonable Tasks		
	*B*	*SE*	*p*	*B*	*SE*	*p*
Step 1:						
(Intercept)	2.56 ***	0.12	<0.001	2.04 ***	0.12	<0.001
Gender ^1^	−0.05	0.13	0.79	0.09	0.13	0.49
Tenure	−0.01	0.004	0.29	−0.00	0.004	0.78
Working hours	0.01	0.01	0.055	0.01	0.01	0.054
Hierarchical level (HL) ^2^	0.05	0.11	0.66	0.11	0.11	0.30
Abusive supervision (AS)	0.64 ***	0.07	<0.001	0.62 ***	0.07	<0.001
*R* ^2^	0.273			0.275		
Step 2:						
(Intercept)	2.56 ***	0.12	<0.001	2.09 ***	0.11	<0.001
Gender^1^	−0.04	0.13	0.75	0.09	0.12	0.45
Tenure	−0.004	0.004	0.27	−0.001	0.004	0.73
Working hours	0.01	0.01	0.056	0.01	0.01	0.054
Hierarchical level (HL) ^2^	0.06	0.11	0.61	0.13	0.11	0.24
Abusive supervision (AS)	0.70 ***	0.08	<0.001	0.72 ***	0.08	<0.001
HL X AS	−0.16	0.15	0.27	−0.32 *	0.14	0.022
*R* ^2^	0.274			0.286		
Δ*R*^2^	0.001			0.011		

Note: *N* = 268. ^1^ 0 = male; 1 = female. ^2^ 0 = nonsupervisory employees (i.e., lowest hierarchical level); 1 = supervisory employees (i.e., higher hierarchical levels). *B* = unstandardized coefficients; * *p* < 0.05; *** *p* < 0.001.
